# Overexpression of translationally controlled tumor protein ameliorates metabolic imbalance and increases energy expenditure in mice

**DOI:** 10.1038/s41366-021-00821-6

**Published:** 2021-04-30

**Authors:** Yejin Jeon, Ji-Young Choi, Eun-Hwa Jang, Je Kyung Seong, Kyunglim Lee

**Affiliations:** 1 Graduate School of Pharmaceutical Sciences, College of Pharmacy, Ewha Womans University, Seoul, Republic of Korea; 2 Laboratory of Developmental Biology and Genomics, BK21 Plus Program for Advanced Veterinary Science and, Research Institute for Veterinary Science, College of Veterinary Medicine, Interdisciplinary Program for Bioinformatics and Korea Mouse Phenotyping Center, Seoul National University, Seoul, Republic of Korea; 3 Department of Food and Nutrition, Chosun University, Dong-gu, Gwangju Republic of Korea

**Keywords:** Fat metabolism, Fats

## Abstract

**Background/Objectives:**

Translationally controlled tumor protein (TCTP) exhibits numerous biological functions. It has been shown to be involved in the regulation of glucose. However, its specific role in metabolism has not yet been clearly elucidated. Here, we aimed to assess the effect of TCTP overexpression on metabolic tissues and systemic energy metabolism.

**Subjects/Methods:**

We investigated whether TCTP can ameliorate the metabolic imbalance that causes obesity using TCTP-overexpressing transgenic (TCTP TG) mice. The mice were subjected to biochemical, morphological, physiological and protein expression studies to define the role of TCTP in metabolic regulation in response to normal chow diet (NCD) compared to high-fat diet (HFD) conditions, and cold environment.

**Results:**

We found that TCTP TG mice show improved metabolic homeostasis under both of NCD and HFD conditions with simultaneous enhancements in glucose tolerance and insulin sensitivity. In particular, we found coincident increases in energy expenditure with significant upregulation of uncoupling protein 1 (UCP1) in the brown adipose tissue (BAT). Moreover, TCTP overexpressing mice exhibit significantly enhanced adaptive thermogenesis of BAT in response to cold exposure.

**Conclusions:**

Overexpression of TCTP ameliorated systemic metabolic homeostasis by stimulating UCP1-mediated thermogenesis in the BAT. This suggests that TCTP may function as a modulator of energy expenditure. This study suggests TCTP may serve as a therapeutic target for obesity and obesity-associated metabolic disorders including type 2 diabetes.

## Introduction

Obesity is a medical condition characterized by an excessive accumulation of body fat, and it is associated with various metabolic diseases including insulin resistance, type 2 diabetes, hypertension, atherosclerosis, hepatic steatosis, and cancer [1, 2]. Therefore, numerous targets are being explored toward designing a rational therapy for obesity. Among them, the most promising target is the brown adipose tissue (BAT). BAT contains numerous mitochondria that directly consume triglycerides for heat production [2]. Thermogenesis process in the BAT is promoted by sympathetic nervous system (SNS) and is mediated by uncoupling protein 1 (UCP1), a mitochondrial carrier protein which mediates the electron transport chain (ETC)-uncoupled mitochondrial ATP synthesis [3–7]. It has been reported that transgenic mice in which BAT genes are ablated are prone to develop obesity [8]. The activation of BAT thermogenesis in adults is inversely proportional to adiposity, body mass index and glucose profiles, indicating these UCP1-expressing BAT play a significant role in enhancing metabolic homeostasis [9]. Recent studies have demonstrated that BAT also functions as an endocrine organ that releases signaling factors that stimulates energy metabolism [10]. These findings made BAT as a therapeutic target in efforts directed at the prevention or treatment of obesity and related diseases.

Translationally controlled tumor protein (TCTP), also known as p23, histamine releasing factor (HRF), or fortilin, is a ubiquitously expressed, multifunctional protein found in eukaryotes [11]. By interacting with many cellular proteins, TCTP participates in numerous biological processes such as cell growth, proliferation, allergic reactions, anti-apoptosis, calcium-binding activity and microtubule stabilizing activity and various types of cancers [12–18]. TCTP has recently been identified as a glucose-regulated protein that protects pancreatic β**-**cells in hyperglycemic condition [19]. Moreover, TCTP is important for expansion of pancreatic β**-**cell mass during development, and knockout of TCTP in these cells causes hyperglycemia and glucose intolerance [20]. Despite the many significant functions attributed to TCTP, its role in obesity and metabolic disease has not yet been established.

We have long been interested in the multiple biological functions and interactions of TCTP. We demonstrated that TCTP inhibits Na^+^, K^+^-ATPase, causing the accumulation of Na^+^ and enhancement of intracellular Ca^2+^ mobilization [18], which in turn triggers the release of neurotransmitters such as catecholamine including norepinephrine from sympathetic neurons [21]. In addition, it is well known that SNS plays a pivotal role in metabolic homeostatic control and blood pressure [22]. In previous study, we showed that overexpression of TCTP in vascular smooth muscle repressed Na^+^, K^+^-ATPase activity, increased intracellular calcium levels, and led to systemic arterial hypertension [23]. Furthermore, we found that TCTP TG mice exhibit reduced body weight and aggressive behavior characterized by sympathetic activation. Based on these findings, we wondered whether the phenotypic properties observed in TCTP TG mice could have resulted from SNS activation by TCTP overexpression. In this study, we found that TCTP overexpression improves a systemic metabolic status through increasing energy expenditure by UCP1-mediated thermogenesis in mice fed either of normal chow diet (NCD) or high-fat diet (HFD). We believe that the study establishes yet another novel function of TCTP, a crucial regulator for metabolic homeostasis.

## Materials and methods

### Animals

TCTP TG mice were generated by Macrogen (Seoul, Korea) by using the targeting construct pCAGGS-TCTP cDNA containing CMV-IE and chicken β-actin promoter [23]. All animals used in this study were on C57BL/6N background. They were individually housed in a specific pathogen-free (SPF) animal facility and provided water ad libitum and fed a NCD (1314; Altromin) or a HFD (D12492; Research Diets) to generate the HFD-induced obesity model. All animal studies were performed according to the Institutional Animal Care and Use Committee (IACUC) guidelines and were approved by the IACUC of the Ewha Womans University (Permit Number: 16-025). Body weight and food intake were recorded weekly from 4 to 10 weeks. Food efficiency ratio (FER) was calculated by applying the equation: FER = (body weight gain (g)/food intake (g)) × 100. The body composition of WT and TCTP TG was assessed at 10th week by nuclear magnetic resonance (LF90 Minispec, Bruker Corp., Texas, USA).

### Glucose and insulin tolerance tests

Intraperitoneal glucose tolerance test (IP-GTT) was carried out at the completion of the experimental period. After 16 h fasting, the mice were injected intraperitoneally with glucose (1.5 g/kg body weight) and blood glucose was measured from the tail veins with a glucometer (Accu-Check Active, Roche, Mannheim, Germany) at 0, 15, 30, 60, 90, and 120 min. For intraperitoneal insulin tolerance test (IP-ITT), fasting blood glucose was assessed (4 h fast, blood taken from the tail vein) using a glucometer at 0 min. Insulin (1 U/kg body weight) was then injected intraperitoneally and blood glucose level was measured again at regular intervals.

### Metabolic studies

Wild type WT and TCTP TG mice were individually housed with an indirect calorimetry system (CaloSys Calorimetry System, TSE Systems, Inc., Bad Homburg, Germany). Metabolic parameters such as oxygen consumption (VO_2_), carbon dioxide production (VCO_2_), respiratory exchange ratios (RER), and locomotor activity were evaluated 48 h after the adaptation period.

### Determination of leptin, adiponectin and catecholamine levels in plasma

Materials and methods are available in ‘Supplementary Materials and Methods’.

### Biochemical parameters in plasma and hepatic lipids

Materials and methods are available in ‘Supplementary Materials and Methods’.

### Cold exposure test

The WT and TCTP TG mice were housed in cold exposure test cages without food supply and bedding but with free access to water for 5 h at 4 °C. Body temperature of mice was measured at 0, 1, 2, 3, 4 and 5 h using an electronic rectal thermometer (Testo 925 rectal probe, Germany). Body weight was measured immediately before and after the cold exposure. At the end of the experiment, mice were housed at the room temperature for 24 h before sacrifice. All mice were anesthetized with zoletil/rompun. The blood, liver, and adipose tissues were collected, snap-frozen in liquid nitrogen, and stored at −80 °C.

### Preparation of protein extracts and immunoblot analyses

Liver and adipose tissue were rinsed with PBS and frozen in liquid nitrogen. Frozen tissues were ground using a tissuelyser (TissueLyser II, Qiagen, Maryland, USA), lysed in modified RIPA buffer containing protease inhibitor cocktails (Roche, Mannheim, Germany) and phosphatase inhibitor cocktails (Sigma-Aldrich Biotechnology, Missouri, USA). Proteins were electrophoresed in 8–12% sodium dodecyl sulfate-polyacrylamide gel (SDS-PAGE) and transferred to Nitrocellulose membranes (Amersham Bioscience, Germany). The membrane was blocked with Tris-buffered saline in 0.1% tween-20 (TBST) containing 5% bovine serum albumin (BSA) or 5% skim milk for 1 h and incubated with the following primary antibodies at 4 °C overnight. Immunoblots were quantified by densitometry using Image J software. Detailed experimental materials and methods are described in ‘Supplementary Materials and Methods’.

### Histological analysis and immunohistochemistry (IHC) staining

The liver and adipose tissues were paraffin-embedded, sectioned into 5 μm thick, and stained with Hematoxylin and eosin (H&E). IHC staining of the paraffin sections were performed as previously described [24]. The IHC sections were photographed and the area was quantified by color deconvolution vector using Image J software. Detailed experimental materials and methods are described in ‘Supplementary Materials and Methods’.

### Statistical analysis

Experimental results are presented as the mean ± standard error of the mean (SEM) from 5 to 8 mice per group. All data are analyzed using GraphPad^TM^ Prisms software version 8.0 (GraphPad Software Inc., CA, USA). Statistical significance was determined using two-tailed unpaired Student’s *t* test for comparing two groups as indicated in the figure legends. Where specified, two-way repeated ANOVA was used, followed by Bonferroni post hoc tests for selected comparisons. Significant differences between means were defined as *P* < *0.05*.

## Results

### TCTP overexpression improved the body composition of mice fed with NCD

**Fig. 1:**
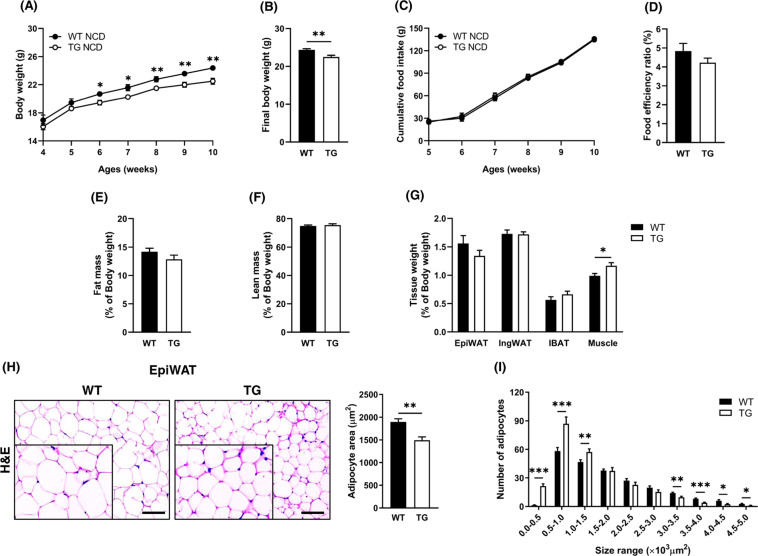
TCTP overexpression improves body composition in NCD-fed mice. **A** Changes in body weight and **B** Final body weight of WT and TCTP TG (*n* = 10). **C** Daily food intake and **D** food efficiency rate (FER) of WT and TCTP TG (*n* = 8). **E** Fat mass and **F** lean mass of 10-week-old WT and TCTP TG (*n* = 6). Masses were measured by LF50 (BRUKER, Germany) and normalized to the body weight. **G** Normalized weights of the EpiWAT, IngWAT, and IBAT, and muscle (*n* = 8). **H** Hematoxylin and Eosin (H&E) staining of EpiWAT and histomorphometric analyses of adipocyte area, and **I** size distribution of EpiWAT ($n = 6 - 7$). The images were magnified by 10X and scale bar is 100 μm. Data are expressed as means ± SEM. Unpaired *t*-test; *P < 0.05, **P < 0.01, ***P < 0.001.

To explore the phenotypes of TCTP TG mice, TCTP TG mice were bred under NCD condition, and phenotypical changes were assessed in comparison with WT mice. The body weight of TCTP TG mice was lower than and became distinguishable from that of WT mice at the age of 6 weeks, and the difference between two groups became more extensive as they were getting aged (Fig. 1A). The final body weight of TCTP TG mice was significantly lower than that of WT mice, whereas the food intake was not different (Fig. 1B, C). Although the differences did not reach the statistical significance, FER, fat mass, and epididymal WAT (EpiWAT) were lower in TCTP TG mice compared to WT mice (Fig. 1D–G). On the other hand, muscle weight was significantly augmented in TCTP TG mice (Fig. 1G). The size of adipocyte in EpiWAT of TCTP TG mice was smaller than that of WT, thus the size distribution shifted toward to small adipocytes in the EpiWAT of TCTP TG (Fig. 1H, I). These results suggest that systemic overexpression of TCTP in mice under NCD condition may ameliorate the body composition through attenuation of the enlargement of adipocytes (hypertrophy) in EpiWAT.

### TCTP overexpression improved plasma lipid profiles, hepatic lipid accumulation, and glucose tolerance

**Fig. 2:**
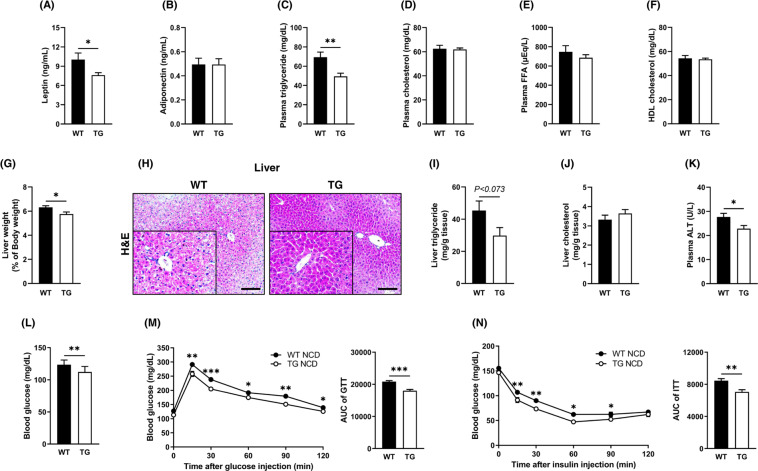
TCTP TG mice fed NCD have improved metabolic homeostasis. **A** Leptin, **B** adiponectin, **C** triglyceride, **D** cholesterol, **E** FFA, and **F** HDL cholesterol levels in plasma of WT and TCTP TG ($n = 5 - 7$). **G** Normalized weight of liver. **H** Triglyceride and **I** cholesterol of the liver, and **J** H&E staining of the liver ($n = 5 - 6$). **K** Plasma ALT level. **L** Blood glucose levels after overnight fast ($n = 5 - 6$). **M** IP-GTT and quantification of the area under the curve (AUC) ($n = 9$). Two-way ANOVA: time effect, genotype effect, *P* < 0.0001. Interaction, *P* < 0.05. Unpaired *t*-test; *P < 0.05, **P < 0.01, ***P < 0.001. **N** IP-ITT and quantification of AUC ($n = 9$). Two-way ANOVA: time effect, *P* < 0.001. Unpaired *t*-test; *P < 0.05, **P < 0.01, ***P < 0.001. The images were magnified by 10X and scale bar is 100 μm. Data are expressed as means ± SEM.

TCTP TG mice exhibited reduced fat mass and significantly reduced hypertrophy of EpiWAT. Therefore, we examined the levels of adipokines such as leptin, adiponectin, and lipid profiles in the plasma. The plasma levels of leptin and triglycerides decreased in TCTP TG mice (Fig. 2A–F). Histomorphometric analysis demonstrated that decrease in the liver weight found in TCTP TG was correlated with attenuated hepatic lipid droplet accumulation and reduced liver triglyceride (Fig. 2G–J). In addition, the plasma ALT level, an indicator of the liver damage, was decreased in TCTP TG mice (Fig. 2K). The results from fasting blood glucose levels, IP-GTT and IP-ITT showed that along with enhanced basal glucose tolerance, both of glucose tolerance and insulin resistance were improved in TCTP TG mice (Fig. 2L–N).

### TCTP TG mice showed enhanced overall energy expenditure

**Fig. 3:**
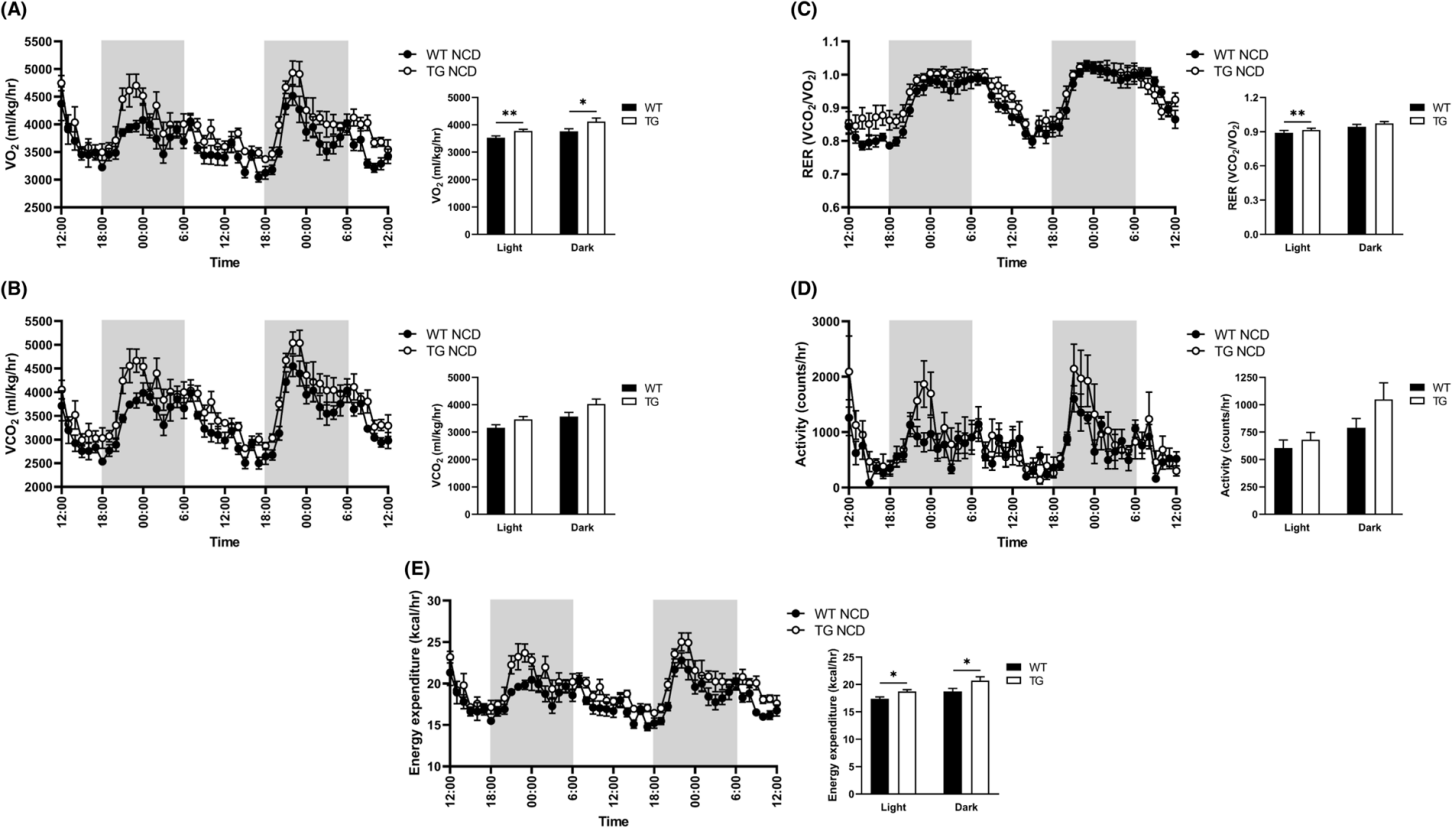
TCTP overexpression enhances energy expenditure in NCD-fed mice. **A** Real-time monitoring curves of VO_2_, **B** VCO_2_, **C** RER, **D** locomotor activity, and **E** energy expenditure of 10-week-old WT and TCTP TG. All bar graphs represent the average values of dark and light cycles. Mice were placed in metabolic cage for 24 h and metabolic parameters were recorded for 48 h ($n = 6$). Data are mean ± SEM. Unpaired *t*-test^;^ *P < 0.05, **P < 0.01.

Metabolic parameters including oxygen consumption, carbon dioxide production, energy expenditure, and physical activity of TCTP TG and WT mice were assessed to compare their metabolic capabilities. Interestingly, indirect calorimetry analysis using the comprehensive laboratory animal monitoring system (CLAMS) showed that the oxygen consumption (VO_2_) and energy expenditure were significantly augmented in TCTP TG group during both at day and night times compared to WT mice (Fig. 3A, E). The carbon dioxide production (VCO_2_) and activity were similar in both groups (Fig. 3B, D). Although RER, which indicates the type of fuel being used for energy production by presenting the ratio of carbon dioxide produced per consumed oxygen during rest or mild aerobic exercise [25, 26], was increased in TCTP TG mice during day, yet both WT and TCTP TG mice used similar type of fuel during day and night (Fig. 3C). Overall, the result shows that TCTP TG mice uses similar type of fuel with WT mice but uses more energy.

### Interscapular BAT (IBAT) thermogenesis was enhanced in TCTP TG mice

**Fig. 4:**
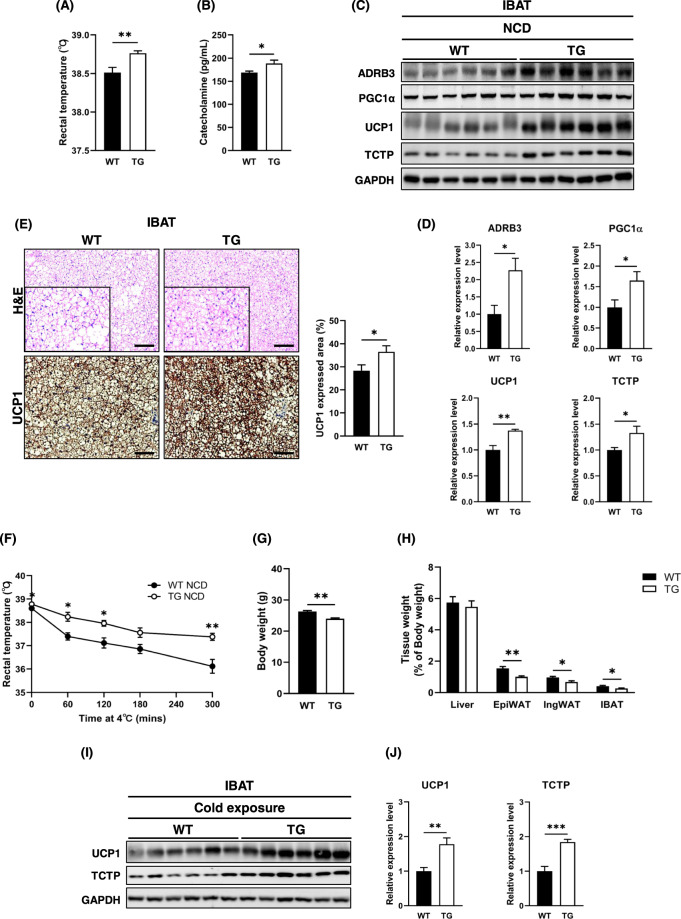
TCTP overexpression activates BAT thermogenesis and enhances resistance to cold in NCD-fed mice. **A** Rectal temperature of 10-week-old WT and TCTP TG ($n = 8$). **B** Plasma levels of catecolamine ($n = 6 - 7$). **C** Immunoblots of ADRB3, PGC1α, UCP1 and TCTP in IBAT. **D** Quantification of expression levels of immunoblotted proteins ($n = 6$). **E** H&E staining and UCP1-IHC of IBAT and quantification of UCP1 stained area (%) ($n = 5 - 6$). The images were magnified by 10X and scale bar is 100 μm. **F** Changes in rectal temperature during 6 h of cold exposure at 4 °C. **G** The body weights of WT and TCTP TG. The body weights were measured 12 h after the cold exposure. Normalized weights of (**H**) liver, EpiWAT, IngWAT, and IBAT ($n = 5 - 6$). **I** Immunoblots of UCP1 and TCTP in IBAT. **J** Quantification of normalized protein expression levels of immunoblot in **I** ($n = 6$). Data are mean ± SEM. Unpaired *t*-test; *P < 0.05, **P < 0.01, ***P < 0.001.

As a portion of daily energy expenditure is required for maintaining the body temperature and catecholamine is known to promote energy expenditure, rectal temperature, and plasma catecholamine of TCTP TG mice were measured. In regard to increased energy expenditure in TCTP TG mice, rectal temperature and the level of plasma catecholamine were also increased. (Fig. 4A, B). We measured the level of UCP1 in the IBAT of TCTP TG mice because UCP1-mediated thermogenesis of BAT is known to respond to sympathetic tone. As with increased plasma catecholamine in TCTP TG mice, protein levels of β3-adrenergic receptor (ADRB3), peroxisome proliferator-activated receptor gamma coactivator 1-alpha (PGC1α), and UCP1 were significantly elevated in the IBAT of TCTP TG mice (Fig. 4C, D). Importantly, histomorphometric analysis of IBAT demonstrated that the protein expression of UCP1 was increased by 22.4% and fewer lipid droplets were found in the IBAT of TCTP TG mice (Fig. 4E). Therefore, it appears that the overexpression of TCTP promotes increase in energy expenditure via UCP1-mediated thermogenesis of IBAT.

### TCTP overexpression augments adaptive thermogenesis of IBAT in response to cold exposure

Adaptive thermogenesis is a type of sympathetic response to cold environment in BAT of rodents, we placed groups of WT and TCTP TG mice into hypothermic condition. TCTP TG mice seemed to be more able to withstand the cold environment, and significant body weight difference between two groups was observed after the cold exposure (Fig. 4F, G). The weights of metabolic tissues, EpiWAT, inguinal WAT (IngWAT), and IBAT of TCTP TG mice were significantly lower than those of WT mice (Fig. 4H). Therefore, we assessed the expression of UCP1 in the IBAT while both groups were being exposed to the cold environment. Not surprisingly, TCTP TG mice had higher UCP1 expression level compared to WT mice (Fig. 4I, J). Hence, it appears that the overexpression of TCTP enhances UCP1-mediated adaptive thermogenesis of IBAT in response to cold environment through increasing the energy expenditure.

### TCTP TG mice were resistant to HFD-induced obesity and metabolic disorders

**Fig. 5:**
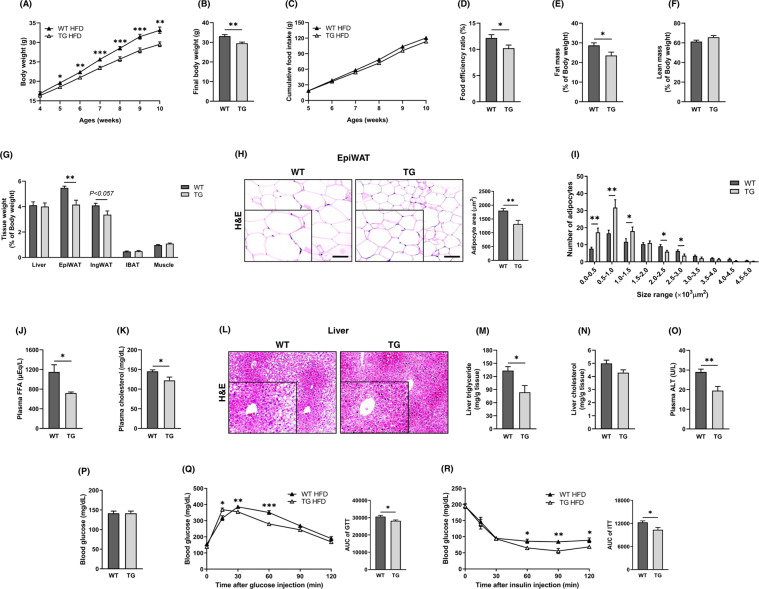
TCTP overexpression improves systemic metabolic homeostasis in mice with HFD-induced obesity. **A** Changes in body weight and **B** Final body weight of WT and TCTP TG ($n = 10$). **C** Daily food intake and **D** food efficiency rate (FER) of WT and TCTP TG ($n = 10$). **E** Fat mass and **F** lean mass of 10-week-old WT and TCTP TG ($n = 6$). Masses were measured by LF50 (BRUKER, Germany) and normalized to the body weight. **G** Normalized weights of the liver, EpiWAT, IngWAT, IBAT and muscle ($n = 6$). **H** H&E staining of EpiWAT and histomorphometric analyses of adipocyte area, and **I** size distribution of EpiWAT ($n = 6 - 7$). The images were magnified by 10X and scale bar is 100 μm. **J** FFA level and **K** cholesterol in plasma of WT and TCTP TG ($n = 5 - 7$). **L** H&E staining of the liver. ($n = 5 - 6$). **M** Triglyceride and **N** cholesterol of the liver, and **O** plasma ALT level. **P** Blood glucose levels after overnight fast ($n = 6$). **Q** IP-GTT and quantification of the area AUC ($n = 5$). Two-way ANOVA: time effect, *P* < 0.0001. Interaction, *P* < 0.05. Unpaired *t*-test; *P < 0.05, **P < 0.01, ***P < 0.001. ***R*** IP-ITT and quantification of AUC ($n = 5$). Two-way ANOVA: time effect, *P* < 0.0001. Unpaired *t*-test; *P < 0.05, **P < 0.01. The images were magnified by 10X and scale bar is 100 μm. Data are mean ± SEM. Unpaired *t*-test; *P < 0.05, **P < 0.01, ***P < 0.001.

The phenotypic differences between WT mice and TCTP TG mice under HFD condition were assessed to see if overexpression of TCTP is able to prevent or ameliorate obesity. The results showed that the phenotypic differences found in HFD-fed animals were generally appeared to be similar with mice fed NCD. On the other hand, more prominent differences were noted in the body weight, food efficiency, fat mass, and weight of EpiWAT between WT and TCTP TG mice under HFD condition (Figs. 5A–G and 1A–G). The difference in food efficiency was worthy of note because it explained how HFD-fed TCTP TG mice gained less weight under isoenergetic condition (Fig. 5D). In the EpiWAT of TCTP TG, the average size of adipocytes was decreased and the size distribution was shifted towarding to smaller adipocytes. These results were congruent to the result of reduced weight of EpiWAT. Also, the weight of IngWAT was decreased. Altogether, the weights of WAT tissues were decreased in TCTP TG mice, and this led to reduced fat mass in TCTP TG mice (Fig. 5E, G, H and I). Thus, overexpression of TCTP seems to attenuate the adipocyte hypertrophy as well as to increase the number of adipocytes (Hyperplasia). As with NCD-fed TCTP TG mice, lipid profiles and adipokines levels were improved in HFD-fed TCTP TG mice (Supplementary Fig. 1). Plasma FFA and the cholesterol levels were significantly reduced (Fig. 5J, K). Hepatic tissue morphology studies showed decreased accumulation of hepatic lipid droplets in the TCTP TG group (Fig. 5L). Consistently, the hepatic triglyceride and cholesterol contents in TCTP TG mice were significantly lower than in WT mice (Fig. 5M, N). Also, ALT level was greatly improved in TCTP TG mice (Fig. 5O). Although, fasting blood glucose level was not different between two groups, the glucose tolerance and insulin resistance were significantly improved in HFD-fed TCTP TG mice with ameliorated hepatic steatosis (Fig. 5P–R). Altogether, the phenotypes of WT and TCTP TG mice, fed with HFD, were clearly distinct. Collectively, these phenotypic differences suggest that overexpression of TCTP confers resistance to HFD-induced obesity and its complications in mice.

### Overexpression of TCTP increased energy expenditure and IBAT thermogenesis through activation of cyclic AMP-responsive element-binding protein (CREB) under HFD condition

**Fig. 6:**
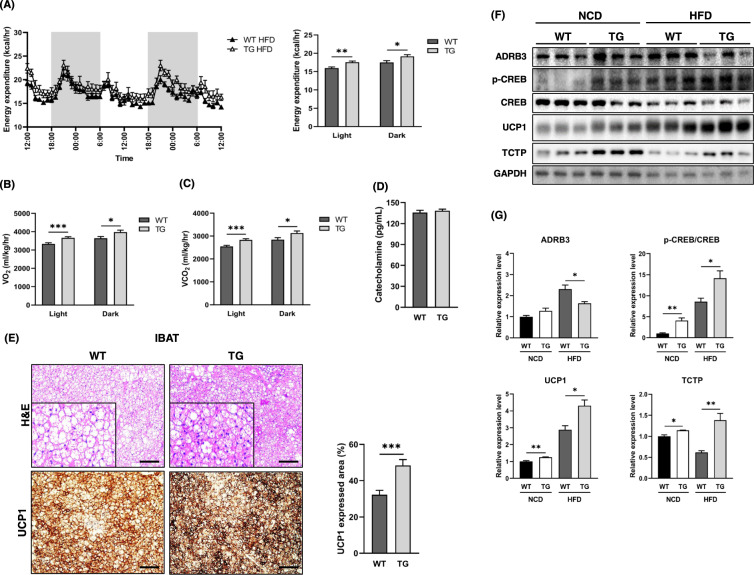
TCTP TG mice fed HFD exhibit enhanced energy expenditure through activation of BAT. **A** Real-time monitoring curves of energy expenditure **B** VO_2_, and **C** VCO_2_ of 10-week-old WT and TCTP TG. All bar graphs represent the average values of dark and light cycles. Mice were placed in metabolic cage for 24 h and metabolic parameters were recorded for 48 h ($n = 6$). **D** Plasma levels of catecolamine. **E** H&E staining and UCP1-IHC of IBAT and quantification of UCP1 stained area (%) ($n = 6$). **F** Immunoblots of ADRB3, p-CREB, CREB, UCP1 and TCTP in IBAT. **G** Quantification of normalized protein expression levels of immunoblot in **F** ($n = 6$). The images were magnified by 10X and scale bar is 100 μm. Data are expressed as means ± SEM. Unpaired *t*-test; *P < 0.05, **P < 0.01, ***P < 0.001.

As with NCD-fed TCTP TG mice, upregulated energy expenditure was maintained in HFD-fed TCTP TG mice both during day and night phases (Fig. 6A). The activity in HFD-fed TCTP TG mice showed statistically insignificant increase over that in WT (Supplementary Fig. 2). Particularly, oxygen consumption and carbon dioxide production were also higher than those in WT mice (Fig. 6B, C). We, then, examined whether the higher UCP1 expression observed in NCD-fed TCTP TG mice remains even after HFD feeding. Consistently, H&E staining and UCP1-IHC of IBAT showed smaller multilocular lipid droplet accumulation and significantly increased expression of UCP1 in HFD-fed TCTP TG (Fig. 6E). Catecholamine, ADRB3, cAMP, PKA, and CREB are major molecules that involve in UCP1 upregulation in the BAT [27–30]. Although plasma catecholamine level was not different between HFD-fed groups and protein level of ADRB3 was unexpectedly decreased in the IBAT of HFD-fed TCTP TG mice compared to HFD-fed WT mice, protein levels of phosphorylated CREB and UCP1 were significantly increased in both of NCD-fed and HFD-fed TCTP TG mice compared to their controls (Fig. 6D–G). The result demonstrated that it seemed that overexpression of TCTP promotes upregulation of UCP1 in the IBAT through both of catecholamine-ADRB3 and cAMP-PKA-CREB pathway under NCD condition whereas it promotes the upregulation only through CREB activation under HFD condition.

## Discussion

The pivotal role of TCTP in the regulation of metabolic homeostasis via energy expenditure in HFD-induced obesity has never been reported. In this paper, we revealed that the overexpression of TCTP may be able to enhance energy expenditure via UCP1-dependent BAT thermogenesis. Although numerous studies have recently reported about certain genes that can activate BAT thermogenesis, here we as a first time report the relation of UCP1 activation in TCTP-induced signaling. We believe that this study provides new insights into the regulation of metabolic homeostasis via TCTP.

To understand the mechanisms underlying the effects of TCTP in metabolic homeostasis, we firstly assessed the phenotype differences between chow-diet fed TCTP TG and WT littermates. We found that the body composition, including muscle and EpiWAT, was improved in the TCTP TG mice compared to WT mice, with simultaneous reductions in plasma triglyceride and leptin levels. Moreover, decreased hepatic lipid accumulation in TCTP TG mice seemed to contribute to improved glucose homeostasis. High levels of energy expenditure are commonly related to decrease in energy storage and protective responses to systemic metabolic damage. Therefore, we examined whether these metabolic improvements in TCTP TG mice could result from increased energy expenditure. As expected, CLAMS indicated that TCTP TG mice exhibited increased energy expenditure during both day and night phases compared with WT mice. Together, these results indicate that metabolic improvements were followed by enhanced energy expenditure in TCTP TG mice.

Total energy expenditure is the sum of energy utilization during external physical activity and internal heat production. Hence, to determine whether TCTP regulates energy expenditure through heat production, we measured the rectal temperatures in TCTP TG and WT controls. In line with the increased energy expenditure, the rectal temperatures of TCTP TG mice were higher than those of WT controls. Consequently, we speculated that the elevated metabolism found in TCTP TG mice was most likely caused by increased thermogenesis, which is associated with BAT activation. This study showed that BAT activation can be mediated by overexpression of TCTP since the IBAT of TCTP TG mice had more intense color and smaller size of the multilocular lipid droplets. In addition, IBAT of TCTP TG mice showed elevated UCP1 staining compared with WT mice.

BAT thermogenesis is regulated by SNS-dependent activation of β-adrenergic receptors (ADRBs) [29]. ADRB3 on the cell membrane of BAT is activated by the SNS stimulation, and the signaling further activates intracellular adenylyl cyclase that converts ATP into cAMP. Then, cAMP promotes the upregulation of UCP1, a thermogenin found on the inner membrane of mitochondria, through stimulating phosphorylation of CREB [28, 30]. In addition, BAT is also known to consume energy in response to excessive energy and cold environment. The sympathetic activation leads to mobilization of fatty acids from WAT, which are then utilized by BAT to dissipate energy as heat [31]. Especially, systemic ADRB3 activation in rodents enhances thermogenic capacity of both brown and beige adipocytes, and it is accompanied by the secretion of catecholamine [2, 32].

Based on this, we examined whether the activation of BAT thermogenesis in TCTP TG mice is caused by SNS stimulation. Interestingly, the overexpression of TCTP upregulated UCP1 and ADRB3 expression. Indeed, the plasma catecholamine level of TG mice was significantly elevated, suggesting the activation of SNS in TCTP TG mice. Consequently, TCTP can be a crucial regulator for UCP1 expression in the BAT by facilitating the modulation of multiple metabolic responses including thermogenesis via SNS activation.

In addition to BAT, muscle is another important tissue responsible for energy expenditure. In this study, NCD-fed TCTP TG mice exhibited increased muscle weight relative to body weight. It has been previously shown that TCTP overexpression protects skeletal muscle in vivo through inhibiting cellular protein degradation [33]. Also, TCTP was shown to play a pivotal role in muscle hypertrophy through regulating cell survival and apoptosis in myostatin-null mice [34]. Therefore, the above results suggest that TCTP overexpression may enhance energy homeostasis by increasing energy expenditure via augmenting muscle as well as BAT.

The effect of TCTP in the IBAT thermoregulation was further confirmed by cold exposure test. It is well known that exposure to cold temperatures triggers thermogenesis in the BAT via activation of SNS [35]. As found in mice maintained under the NCD condition, TCTP TG mice exhibited enhanced thermogenesis under the cold condition. Particularly, the body weight and the weights of metabolic tissues significantly decreased in TCTP TG mice than in the WT mice after cold exposure. As expected, UCP1 expression also significantly increased in the IBAT of TCTP TG. Taken together, these results demonstrate that TCTP is involved in the regulation of thermogenesis via upregulation of UCP1 in the IBAT and reinforced our idea that the increase in energy expenditure found in TCTP TG mice is at least partially due to BAT activation.

Finally, the metabolic efficiency of TCTP in the obesity was assessed by HFD-induced obesity model. TCTP TG mice showed markedly reduced body weight, fat mass, and food efficiency. Notably, reduced food efficiency in HFD-fed TCTP TG mice explained less weight gain under the isoenergetic condition. Furthermore, TCTP overexpression reduced lipid accumulation in liver and WAT but improved glucose tolerance and insulin sensitivity. Importantly, TCTP TG mice maintained increased energy expenditure and thermoregulation via UCP1 activation even under HFD-fed condition. These results indicate that HFD-induced metabolic imbalance was ameliorated in TCTP TG mice.

As the overexpression of TCTP efficiently ameliorated HFD-induced metabolic imbalance, the upregulation BAT thermogenesis along with the increment in plasma catecholamine level was expected. However, the catecholamine level was unexpectedly the same between HFD-fed TCTP TG and WT groups. Hence, including TCTP, SNS-related protein profiles of the IBAT of WT or TCTP TG mice either fed with NCD or HFD were assessed and compared through western blot analysis. The protein level of ADRB3 was unexpectedly decreased in HFD-fed TCTP mice compared to WT controls, whereas UCP1 and phosphorylated CREB were significantly increased in TCTP TG mice fed either with NCD or HFD. Of note, the difference of TCTP protein expression was greater between HFD-fed TCTP TG and WT mice than that of NCD-fed groups. Accordingly, we speculated that under HFD condition, the overexpression of TCTP may not control UCP1 induced BAT thermogenesis through commonly known extracellular sympathetic stimulus but rather through intracellular activation of CREB.

In a previous study, we demonstrated that overexpression of TCTP triggers intracellular Ca^2+^ influx by repressing Na^+^, K^+^-ATPase [18]. It has also been reported that Ca^2+^ regulates the transcription factor CREB which is activated upon phosphorylation and binds to response elements in the promoter region of many genes including UCP1 [28, 30]. Hence, the overexpression of TCTP may have reciprocal interaction with intracellular upregulation of CREB. It may also indirectly promote activation of CREB signaling through promoting SNS signaling [36]. Consequently, under HFD condition, it seemed that the overexpression of TCTP led to the upregulation of CREB through regulation of Ca^2+^ influx in the IBAT. Whereas, under NCD condition, it seemed that the overexpression of TCTP led to the increase in transcription of and activation of CREB, which may promote upregulation of UCP1 either through direct control or indirectly with TCTP mediated SNS signaling. Under both of feeding conditions, the overexpression of TCTP clearly involves in UCP1-mediated IBAT thermogenesis, but it is complicated by its mode of action and by diet conditions. In addition, it also has been reported that there is an interrelation between TCTP and CREB in vivo, that the activation of CREB signaling may increase the expression of TCTP [37]. It suggests both TCTP and UCP1 may also be regulated in parallel by CREB pathway. Therefore, further studies are needed to determine an interrelation between TCTP and CREB.

These findings do not exclude the possibility that TCTP could stimulate energy expenditure through other mechanisms in addition to the activation of BAT. Nevertheless, it is clear that TCTP contributes to the amelioration of systemic metabolic imbalance through enhancing energy expenditure by UCP1 activation in the BAT. Taken together, the present findings suggest TCTP as a potential target for treatment of obesity and its related metabolic disorders.

## Supplementary information

Suppementary data

## References

[1] Spiegelman BM, Flier JS (2001). Obesity and the regulation of energy balance. Cell.

[2] Lowell BB, Spiegelman BM (2000). Towards a molecular understanding of adaptive thermogenesis. Nature.

[3] Cinti S (2005). The adipose organ. Prostaglandins Leukot Essent Fatty Acids.

[4] Ghorbani M, Claus TH, Himms-Hagen J (1997). Hypertrophy of brown adipocytes in brown and white adipose tissues and reversal of diet-induced obesity in rats treated with a beta3-adrenoceptor agonist. Biochem Pharmacol.

[5] Berbee JF, Boon MR, Khedoe PP, Bartelt A, Schlein C, Worthmann A (2015). Brown fat activation reduces hypercholesterolaemia and protects from atherosclerosis development. Nat Commun.

[6] Labbe SM, Caron A, Bakan I, Laplante M, Carpentier AC, Lecomte R (2015). In vivo measurement of energy substrate contribution to cold-induced brown adipose tissue thermogenesis. FASEB J.

[7] Chondronikola M, Volpi E, Borsheim E, Porter C, Annamalai P, Enerback S (2014). Brown adipose tissue improves whole-body glucose homeostasis and insulin sensitivity in humans. Diabetes.

[8] Lowell BB, Susulic VS, Hamann A, Lawitts JA, Himms-Hagen J, Boyer BB (1993). Development of obesity in transgenic mice after genetic ablation of brown adipose tissue. Nature.

[9] Cypess AM, Lehman S, Williams G, Tal I, Rodman D, Goldfine AB (2009). Identification and importance of brown adipose tissue in adult humans. N Engl J Med.

[10] Villarroya J, Cereijo R, Villarroya F (2013). An endocrine role for brown adipose tissue?. Am J Physiol Endocrinol Metab.

[11] Yenofsky R, Bergmann I, Brawerman G (1982). Messenger RNA species partially in a repressed state in mouse sarcoma ascites cells. Proc Natl Acad Sci U S A.

[12] Hsu YC, Chern JJ, Cai Y, Liu MY, Choi KW (2007). Drosophila TCTP is essential for growth and proliferation through regulation of dRheb GTPase. Nature.

[13] Yarm FR (2002). Plk phosphorylation regulates the microtubule-stabilizing protein TCTP. Mol Cell Biol.

[14] Arcuri F, Papa S, Carducci A, Romagnoli R, Liberatori S, Riparbelli MG (2004). Translationally controlled tumor protein (TCTP) in the human prostate and prostate cancer cells: expression, distribution, and calcium binding activity. Prostate.

[15] Bommer UA, Vine KL, Puri P, Engel M, Belfiore L, Fildes K et al. Translationally controlled tumour protein TCTP is induced early in human colorectal tumours and contributes to the resistance of HCT116 colon cancer cells to 5-FU and oxaliplatin. Cell Commun Signal. 2017;15:9. 10.1186/s12964-017-0164-3 PMC5286767 28143584

[16] Kaarbo M, Storm ML, Qu S, Waehre H, Risberg B, Danielsen HE et al. TCTP is an androgen-regulated gene implicated in prostate cancer. Plos One. 2013;8:e69398. 10.1371/journal.pone.0069398 PMC3718683 23894469

[17] MacDonald SM, Rafnar T, Langdon J, Lichtenstein LM (1995). Molecular identification of an IgE-dependent histamine-releasing factor. Science.

[18] Jung J, Ryu S, Ki IA, Woo HA, Lee K. Some biological consequences of the inhibition of Na,K-ATPase by translationally controlled tumor protein (TCTP). Int J Mol Sci. 2018;19:1657. 10.3390/ijms19061657 PMC6032315 29867020

[19] Diraison F, Hayward K, Sanders KL, Brozzi F, Lajus S, Hancock J (2011). Translationally controlled tumour protein (TCTP) is a novel glucose-regulated protein that is important for survival of pancreatic beta cells. Diabetologia.

[20] Tsai MJ, Yang-Yen HF, Chiang MK, Wang MJ, Wu SS, Chen SH (2014). TCTP is essential for beta-cell proliferation and mass expansion during development and beta-cell adaptation in response to insulin resistance. Endocrinology.

[21] Neher E, Sakaba T (2008). Multiple roles of calcium ions in the regulation of neurotransmitter release. Neuron.

[22] Goran MI (2000). Energy metabolism and obesity. Med Clin North Am.

[23] Kim MJ, Kwon JS, Suh SH, Suh JK, Jung J, Lee SN (2008). Transgenic overexpression of translationally controlled tumor protein induces systemic hypertension via repression of Na+,K+-ATPase. J Mol Cell Cardiol.

[24] Sheverdin V, Jung J, Lee K (2013). Immunohistochemical localization of translationally controlled tumor protein in the mouse digestive system. J Anat.

[25] Pendergast DR, Leddy JJ, Venkatraman JT (2000). A perspective on fat intake in athletes. J Am Coll Nutr.

[26] Simonson DC, DeFronzo RA (1990). Indirect calorimetry: methodological and interpretative problems. Am J Physiol.

[27] Nedergaard J, Golozoubova V, Matthias A, Asadi A, Jacobsson A, Cannon B (2001). UCP1: the only protein able to mediate adaptive non-shivering thermogenesis and metabolic inefficiency. Biochim Biophys Acta.

[28] Yin JC, Wallach JS, Del Vecchio M, Wilder EL, Zhou H, Quinn WG (1994). Induction of a dominant negative CREB transgene specifically blocks long-term memory in Drosophila. Cell.

[29] Bachman ES, Dhillon H, Zhang CY, Cinti S, Bianco AC, Kobilka BK (2002). betaAR signaling required for diet-induced thermogenesis and obesity resistance. Science.

[30] Rim JS, Xue B, Gawronska-Kozak B, Kozak LP (2004). Sequestration of thermogenic transcription factors in the cytoplasm during development of brown adipose tissue. J Biol Chem.

[31] Guarino D, Nannipieri M, Iervasi G, Taddei S, Bruno RM (2017). The role of the autonomic nervous system in the pathophysiology of obesity. Front Physiol.

[32] Saito M (2013). Brown adipose tissue as a regulator of energy expenditure and body fat in humans. Diabetes Metab J.

[33] Goodman CA, Coenen AM, Frey JW, You JS, Barker RG, Frankish BP (2017). Insights into the role and regulation of TCTP in skeletal muscle. Oncotarget.

[34] Chelh I, Meunier B, Picard B, Reecy MJ, Chevalier C, Hocquette JF (2009). Molecular profiles of Quadriceps muscle in myostatin-null mice reveal PI3K and apoptotic pathways as myostatin targets. BMC Genomics.

[35] Cannon B, Nedergaard J (2004). Brown adipose tissue: function and physiological significance. Physiol Rev.

[36] Andree H, Thiele H, Fahling M, Schmidt I, Thiele BJ (2006). Expression of the human TPT1 gene coding for translationally controlled tumor protein (TCTP) is regulated by CREB transcription factors. Gene.

[37] Altarejos JY, Montminy M (2011). CREB and the CRTC co-activators: sensors for hormonal and metabolic signals. Nat Rev Mol Cell Biol.

