# Supplementary material for: Overexpression of translationally controlled tumor protein ameliorates metabolic imbalance and increases energy expenditure in mice
Source: Int J Obes (Lond). 2021 Apr 30;45(7):1576–87. doi: 10.1038/s41366-021-00821-6 (PMC8236403; doi:10.1038/s41366-021-00821-6)
Supplement: Supplementary file 1 — Suppementary data [file 41366_2021_821_MOESM1_ESM.docx]

**Supplementary Materials and Methods**

**Determination of Leptin, Adiponectin and Catecholamine Levels in Plasma**

Plasma concentrations of adipokines (leptin and adiponectin) and catecholamine were measured using two different ELISA kits (Crystal Chem, Illinois, USA, and BioVision, Inc., California, USA) by following manufacturer’s instructions.

**Biochemical Parameters in Plasma and Hepatic Lipids**

Alanine aminotransferase (ALT), total cholesterol, high-density lipoprotein (HDL) cholesterol, triglyceride, and free fatty acids (FFA) in plasma were determined by Global Clinical Central Lab (GC Pharm Corporation, Gyeonggi-do, Korea). Hepatic lipid was extracted using a lipid extraction kit (BioVision, Inc., California, USA), and hepatic cholesterol and triglyceride were measured by quantification kits (ASAN Pharm, Seoul, Korea).

**Preparation of Protein Extracts and Immunoblot Analyses**

Liver and adipose tissue were rinsed with PBS and frozen in liquid nitrogen. Frozen tissues were ground using a tissuelyser (TissueLyser II, Qiagen, Maryland, USA), lysed in modified RIPA buffer containing protease inhibitor cocktails (Roche, Mannheim, Germany) and phosphatase inhibitor cocktails (Sigma-Aldrich Biotechnology, Missouri, USA). Proteins were electrophoresed in 8-12% sodium dodecyl sulfate-polyacrylamide gel (SDS-PAGE) and transferred to Nitrocellulose membranes (Amersham Bioscience, Germany). The membrane was blocked with Tris-buffered saline in 0.1% tween-20 (TBST) containing 5% bovine serum albumin (BSA) or 5% skim milk for 1 hour and incubated with the following primary antibodies: TCTP, UCP1, β3-adrenergic receptor (ADRB3), cyclic AMP-responsive element-binding protein (CREB), phospho-CREB (p-CREB), peroxisome proliferator-activated receptor gamma coactivator 1-alpha (PGC1α) (Abcam, Cambridge, UK); glyceraldehyde-3-phosphate dehydrogenase (GAPDH) (Cell Signaling Technology, Massachusetts, USA); in TBST containing 5% BSA at 4℃ overnight. After wash with TBST, membrane was incubated with HRP-conjugated secondary antibody for 1 h at room temperature. And the blots were visualized by enhanced chemiluminescence (ECL prime Western blotting detection system, Amersham Bioscience, Germany). Immunoblots were quantified by densitometry using Image J software.

**Histological Analysis and Immunohistochemistry (IHC) Staining**

The whole blood was collected via cardiac puncture, transcardially perfused with phosphate-buffered saline (PBS). The liver and adipose tissues were paraffin-embedded, sectioned into 5 μm thick, and stained with Hematoxylin and eosin (H&E). IHC staining of the paraffin sections were performed as previously described ^24^. Briefly, the slides were incubated with primary anti-TCTP antibody (1:500, ab37506) and anti-UCP1 antibody (1:1000, ab10983) at 4℃ overnight then with secondary anti-rabbit IgG conjugated with peroxidase using a ImmPRESS Reagent Kit (Vector Laboratories, San Francisco, USA) for 1 hour at room temperature followed by detection with 0.05% 3,3'-Diaminobenzidine (Sigma-Aldrich Biotechnology, Missouri, USA) solution with hydrogen peroxide. Slides were viewed under bright-field optical microscopy using a light microscope (Axio Scope. A1, Zeiss, Oberkochen, Germany), and representative sections were photographed. The IHC sections were photographed and the area was quantified by color deconvolution vector using Image J software.

**Supplementary Data**


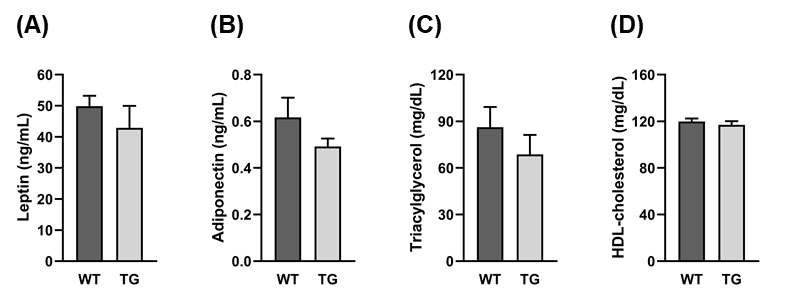


**Supplementary Figure 1. Lipid profiles of HFD-fed WT and TCTP TG mice.**

(**A**) Leptin, (**B**) adiponectin, (**C**) triglyceride, and (**D**) HDL cholesterol levels in plasma of WT and TCTP TG ($n=5-7$).


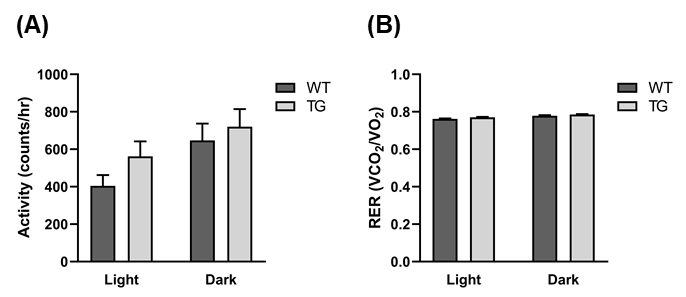


**Supplementary Figure 2. RER and locomotor activity of WT and TCTP TG mice under HFD condition.**

(**A**) RER, (**B**) locomotor activity of 10-week-old WT and TCTP TG. All bar graphs represent the average values of dark and light cycles. Mice were placed in metabolic cage for 24 hours and metabolic parameters were recorded for 48 hours ($n=6$). Data are mean ± SEM. Unpaired t-test; P^*^ < 0.05, P^**^ < 0.01.
